# Increased CD86 but Not CD80 and PD-L1 Expression on Liver CD68^+^ Cells during Chronic HBV Infection

**DOI:** 10.1371/journal.pone.0158265

**Published:** 2016-06-27

**Authors:** Elias A. Said, Iman Al-Reesi, Marwa Al-Riyami, Khalid Al-Naamani, Shadia Al-Sinawi, Mohammed S. Al-Balushi, Crystal Y. Koh, Juma Z. Al-Busaidi, Mohamed A. Idris, Ali A. Al-Jabri

**Affiliations:** 1 Department of Microbiology and Immunology, College of Medicine and Health Sciences, Sultan Qaboos University, P.O. Box: 35, Code: 123, Muscat, Oman; 2 Department of Pathology, College of Medicine and Health Sciences, Sultan Qaboos University, P.O. Box: 35, Code: 123, Muscat, Oman; 3 Department of Medicine, Armed Forces Hospital, Muscat, Oman; Institut Pasteur, FRANCE

## Abstract

**Background:**

The failure to establish potent anti-HBV T cell responses suggests the absence of an effective innate immune activation. Kupffer cells and liver-infiltrating monocytes/macrophages have an essential role in establishing anti-HBV responses. These cells express the costimulatory molecules CD80 and CD86. CD80 expression on antigen-presenting cells (APCs) induces Th1 cell differentiation, whereas CD86 expression drives the differentiation towards a Th2 profile. The relative expression of CD80, CD86 and PD-L1 on APCs, regulates T cell activation. Few studies investigated CD80 and CD86 expression on KCs and infiltrating monocytes/macrophages in HBV-infected liver and knowledge about the expression of PD-L1 on these cells is controversial. The expression of these molecules together in CD68^+^ cells has not been explored in HBV-infected livers.

**Methods:**

Double staining immunohistochemistry was applied to liver biopsies of HBV-infected and control donors to explore CD80, CD86 and PD-L1 expression in the lobular and portal areas.

**Results:**

Chronic HBV infection was associated with increased CD68^+^CD86^+^ cell count and percentage in the lobular areas, and no changes in the count and percentage of CD68^+^CD80^+^ and CD68^+^PD-L1^+^ cells, compared to the control group. While CD68^+^CD80^+^ cell count in portal areas correlated with the fibrosis score, CD68^+^CD80^+^ cell percentage in lobular areas correlated with the inflammation grade.

**Conclusion:**

The upregulation of CD86 but not CD80 and PD-L1 on CD68^+^ cells in HBV-infected livers, suggests that these cells do not support the induction of potent Th1. Moreover, the expression of CD80 on CD68^+^ cells correlates with liver inflammation and fibrosis.

## Introduction

Close to 240 million people around the world are infected with the hepatitis B virus (HBV) [1]. HBV causes liver diseases that vary in severity from person to person, and 15–40% of infected individuals develop liver cirrhosis with possible progression to liver cancer [2]. In chronic HBV patients the immune system fails to mount and maintain proper HBV-specific T cell responses. Furthermore, HBV-specific T cells are difficult to be detected in the blood and liver, and are functionally impaired [3]. PD-1 is over expressed on HBV-specific T cells, which produce only low amounts of IFN-γ and cannot differentiate into memory cells [4]. A number of factors have been proposed to explain the weak T-cell response, including the impaired function of dendritic cells (DCs) and macrophages [5]. Moreover, the spontaneous clearance of the virus in acute hepatitis was suggested to be due to the action of the innate immune system [6]. Kupffer cells (KCs) and infiltrating monocytes/macrophages constitute the principal population of innate immune cells in the liver [7]. They participate in the immune activation, antiviral immunity and tissue damage associated with HBV infection [7]. They express the co-stimulatory molecules CD80 (B7.1) and CD86 (B7.2), which regulate T cell responses [8]. Both molecules bind to CD28 and CTLA-4 expressed on T cells. CD86 upregulation on APCs occurs before CD80 and CD86 stimulates CD28 before the expression of CD80 that has a higher ability to initiate inhibitory signals through its interaction with CTLA-4 [9,10,11]. On the other hand, CD80 and CD86 might have different roles in regulating the T helper (Th) responses. While CD80 expression on APCs mainly drives T cell differentiation towards a Th1 profile, CD86 leads the differentiation towards a Th2 profile [12,13,14,15,16]. Interestingly, during HBV infection T cell response, especially in liver infiltrating lymphocytes, is associated with the production of IL-10 and Th2 cytokines rather than Th1 cytokines, and Th1 responses are weak in chronic HBV-infected patients when compared with resolver [2,17,18].

APCs including KCs and infiltrating monocytes/macrophages also express PD-L1 and PD-L2 to avoid hyper-activation of the immune system [19]. The levels of PD-L1 and CD80/CD86 signals on APCs may control the magnitude of T cell activation [20,21].

Little is known about KCs role in HBV pathogenesis [7]. To our knowledge only one study has investigated the expression of CD80 and CD86 on KCs in HBV infection. This study found that only few KCs express these molecules [22]. The information about the changes in PD-L1 expression on KCs during HBV infection is controversial [23,24,25,26].

In this study double staining immunohistochemistry is used for the first time to explore the differences in the expression of CD80 and CD86 together with PD-L1 in CD68^+^ cells in the lobular and portal areas of the liver, and the correlation of their expression with the fibrosis score and grade of inflammation during HBV infection. This provides information about the potential stimulatory/inhibitory profile of monocytes/macrophages and KCs in the liver due to the balance of the expression of these molecules in HBV-infected patients.

## Methods

### Study population

The study included formalin fixed paraffin wax embedded specimens of liver tissues from 16 chronic hepatitis B-infected patients and 14 HBV^-^ individuals as a control group (Table 1). Specimens were obtained from the Pathology Departments at the Sultan Qaboos University Hospital (SQUH) and the Armed Force Hospital (AFH). Individuals with autoimmune diseases, microbial infection other than HBV, anti-HBV therapy, current ethanol abuse, non-alcoholic steatohepatitis, metabolic liver diseases, drug/toxin induced hepatitis or individuals with established cirrhosis were excluded. All HBV-infected patients included were positive for HBs-Ag and have detectable serum HBV DNA. Individuals included as controls had high levels of liver enzymes of unknown etiology or were liver transplant donors. The control group also included biopsies of unaffected areas for secondary hepatic malignancy (carcinoma). This control group was also used in a study, which was done in parallel, to investigate CD80, CD86 and PD-L1 expression in liver CD68^+^ cells during chronic HCV infection [27]. Since the experimental procedure consisted of staining biopsies that were already done and stored in the bank of the Pathology Department, and did not implicate any special sample (biopsy) collection (article 32 of the Declaration of Helsinki), patients consent was impracticable to obtain. The study and the procedure (the absence of consent) were approved by the Sultan Qaboos University Ethics Committee (MREC#742). The data were analyzed anonymously. To maintain confidentiality, every patient was assigned with a unique identification number.

**Table 1:** Characteristics of the study population. Chronic HBV-infected patients (n = 16) and Control individuals (n = 14) were included in the study.

	Gender (Male/Female)	Mean age (Yr)	Viral load (x10^5^IU/ml)	Fibrosis scores	METAVIR activity score
**Control**	9/5	30.5±9.7	-	1.2±1.5	0.43±0.9
**HBV-infected**	10/6	37±10.2	5.5 ± 8.2	1.5±0.96	1.1±0.99

M, male; F, female; Yr, Year; VL, viral load

### Immunohistochemistry (IHC) and Identification of cells

Liver-infiltrating monocytes/macrophages and KCs were identified based on their morphology and CD68 expression. To detect the expression of PD-L1, CD80 or CD86 on CD68^+^ cells, liver sections were double-stained using the double staining kit [Polink DS-MR-Hu D2 Kit, IHC, Golden Bridge International Lab (GBI); US] following the manufacturer protocol. Heat-induced epitope retrieval (HIER) was performed using an antigen retrieval buffer pH 9.0 for 20 minutes. Optimal dilutions of the primary antibodies were established by titrating on tonsil tissue. For each set of staining, a tonsil section was included as a positive control whereas a negative control consisted of replacing primary Abs with isotype Ab or antibody diluent. Nonspecific proteins were blocked, then primary Abs were added for 30 min at RT and washed with TBS buffer. Alkaline phosphatase (AP) polymer anti-Mouse IgG was used to detect mouse monoclonal anti-CD68 Ab (Dako, USA) and horseradish peroxidase (HRP) polymer anti-Rabbit IgG was used to detect rabbit monoclonal anti-CD80, CD86 or PD-L1 Abs (Abcam, UK). Two distinct chromogens were used; GBI Permanent-Red (Red color, used with AP polymer anti-Mouse IgG) and Emerald chromogen (Green color, used with HRP polymer anti-Rabbit IgG). Slides were observed under light microscopy (Olympus BX53). CD68^+^cells stained red and the co-localization was observed as a presence of both green (PD-L1, CD80 or CD86) and red (CD68) colors in the same cell. Positive cells were counted in 5 high power fields (diameter of the field = 0.44 mm) with 60x objective and 10x ocular. This was done for both portal and lobular areas. Masson Trichrome stained slides were used to assess stages of fibrosis and grades of inflammation using Metavir scoring system.

### Statistical analysis

The significance of the differences in the cell count and protein expression between different groups was assessed using Mann Whitney test. The significance of the differences in the expression of the three proteins in the same group or the same patients (between two areas) was assessed using Wilcoxon test. The significance of the correlations was assessed using Spearman test. *P* values < 0.05 were considered statistically significant. The results were analyzed using Microsoft Excel, Statistical Package for the Social Sciences (SPSS) version 20 and Prism softwares.

## Results

### CD68^+^ cell count in the liver of HBV infected patients

**Fig 1:**
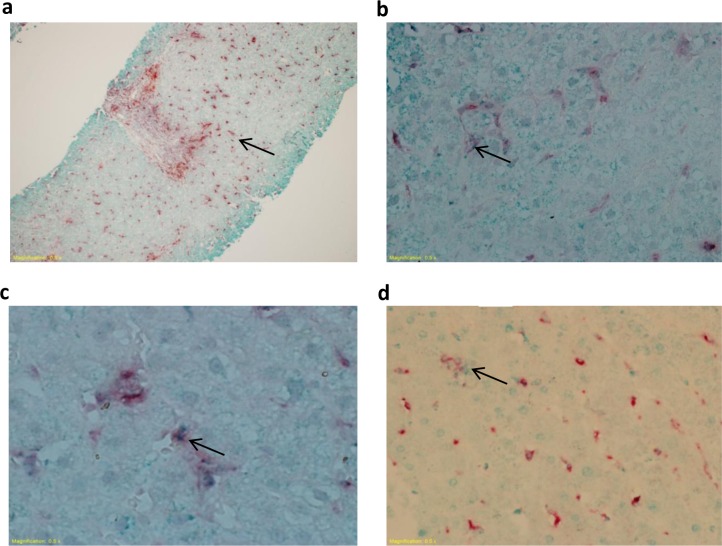
CD68, CD80, CD86 and PD-L1 staining in liver biopsies. Biopsies from HBV-infected and control individuals were stained with mouse anti-human CD68 and rabbit anti-human CD80, CD86 or PD-L1 Abs. Representative photomicrographs showing (**A**) CD68 red staining indicating CD68^+^ cells and (**B-D**) CD80, CD86 or PD-L1 green staining observed as dots within the red stained cells indicating CD68^+^CD80^+^, CD68^+^CD86^+^ and CD68^+^PD-L1^+^ cells. This figure is also used in a related article about the expression of CD80, CD86 and PD-L1 in liver CD68^+^ cells during chronic HCV infection (the same control group was used in both studies that were conducted in parallel) [27].

We identified KCs and liver-infiltrating monocytes/macrophages based on their morphology and CD68 expression. KCs had the form of spindle like cells lining the sinusoids within hepatic lobules. Monocytes/macrophages appeared as round cells in the portal areas. A double staining IHC technique, combining anti-CD68 Ab with anti-CD80, anti-CD86 or anti-PD-L1 Abs, was used to detect CD68^+^ cells and the expression of CD80, CD86 or PD-L1 on their surface, in the lobular and portal areas. The red staining of the cells indicated CD68 expression (Fig 1A), whereas CD80, CD86 or PD-L1 staining was detected as small green dots within the red stained cells (Fig 1B, 1C and 1D respectively).

To determine the effect of HBV infection on CD68^+^ cell number in the liver, we quantified their count upon staining with the anti-CD68 Ab. We found no differences in the count of CD68^+^ cells in the lobular and portal areas between HBV-infected patients and the control group (Table 2).

**Fig 2:**
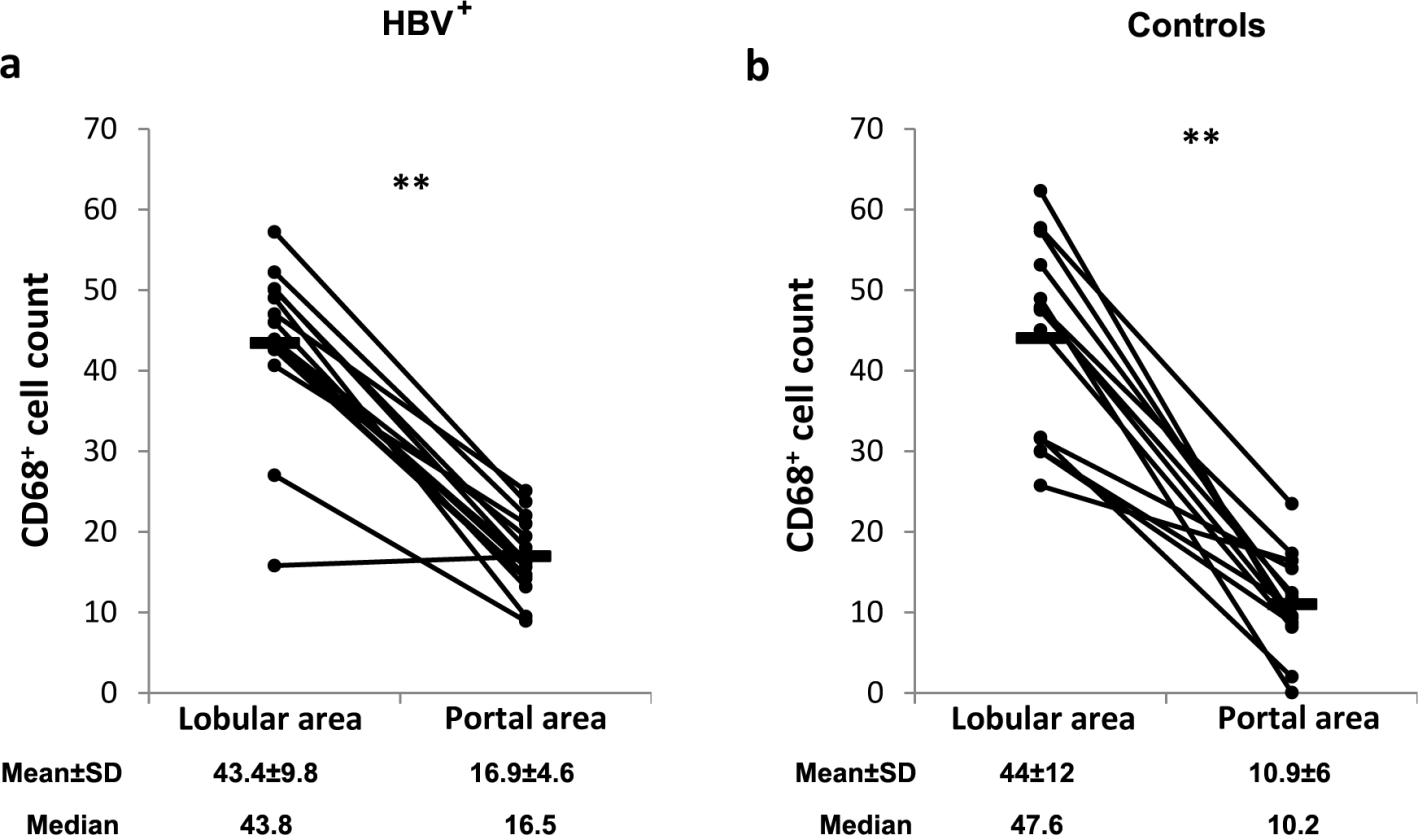
CD68^+^ cell count in the lobular and portal areas of HBV-infected and control livers. Biopsies from HBV-infected and control individuals were stained with mouse anti-human CD68 Ab. Positive cells were counted in 5 high power fields (diameter of the field = 0.44 mm). **A.** CD68^+^ cell count in the lobular and portal areas of HBV-infected livers. **B.** CD68^+^ cell count in the lobular and portal areas of control livers. Panel b was also used in a related article about the expression of CD80, CD86 and PD-L1 in liver CD68^+^ cells during chronic HCV infection because the same control group was used in both studies that were conducted in parallel. * *P* value<0.05. ** *P* value<0.01.

Monocytes/macrophages density differs between the areas of the liver, although these cells are present all over the liver [28]. To investigate the effect of HBV infection on CD68^+^ cells distribution between the lobular and portal areas of the liver, their number was assessed in these areas in HBV-infected and control donors. A significantly higher CD68^+^ cell count was observed in the lobular (cell density = 182.4±41.2 cells/mm^2^ and 184.9±50.6 cells/mm^2^ in patients and controls respectively) areas when compared to the portal areas (cell density = 71.4±19.3 cells/mm^2^ and 46.2±25.2 cells/mm^2^ in patients and controls respectively) in patients and controls (≈3 times higher; *p* = 0.004 and *p* = 0.006 respectively; Fig 2A and 2B).

**Table 2:** Counts of CD68^+^ cells, CD68^+^CD80^+^ cells, CD68^+^CD86^+^ cells and CD68^+^PD-L1^+^ cells. The average count ± S.D. for each areas of the liver is represented as indicated. The *p* value indicates the significance of differences between HBV-infected and control groups.

	Lobular	Portal	Total
	**CD68**^**+**^ **count**		
**HBV-infected patients**	43.4 ± 9.8	17 ± 4.6	60.4 ± 12.2
**Controls**	44 ± 12.04	11 ± 6	55 ± 13.8
***P* value**	0.48	0.355	0.383
	**CD68**^**+**^**CD80**^**+**^ **count**		
**HBV-infected patients**	7.2 ± 9.1	3.5 ± 3.9	12.2 ± 9.8
**Controls**	7.6 ± 11.5	1.8 ± 2.8	9.3 ± 11.9
***P* value**	0.441	0.314	0.308
	**CD68**^**+**^**CD86**^**+**^ **count**		
**HBV-infected patients**	11.6 ± 10.9	1.9 ± 3.2	13.5 ± 11
**Controls**	3.9 ± 4	1.6 ± 2.3	5.5 ± 5.7
***P* value**	0.025	0.67	0.025
	**CD68**^**+**^**PD-L1**^**+**^ **count**		
**HBV-infected patients**	2.2 ± 2	0.7 ± 0.7	2.9 ± 2.5
**Controls**	2.5 ± 2	0.5 ± 0.8	3 ± 2.5
***P* value**	0.632	0.78	0.708

The CD68^**+**^ cell count did not correlate with HBV viral load (*p*>0.05). Moreover, we did not observe any association between the counts of CD68^+^ cells and the age and sex of the individuals in the HBV-infected or the control groups (*p*>0.05).

### Increase in CD68^+^CD86^+^ cell count and percentage in the lobular areas of the liver of HBV-infected patients

**Fig 3:**
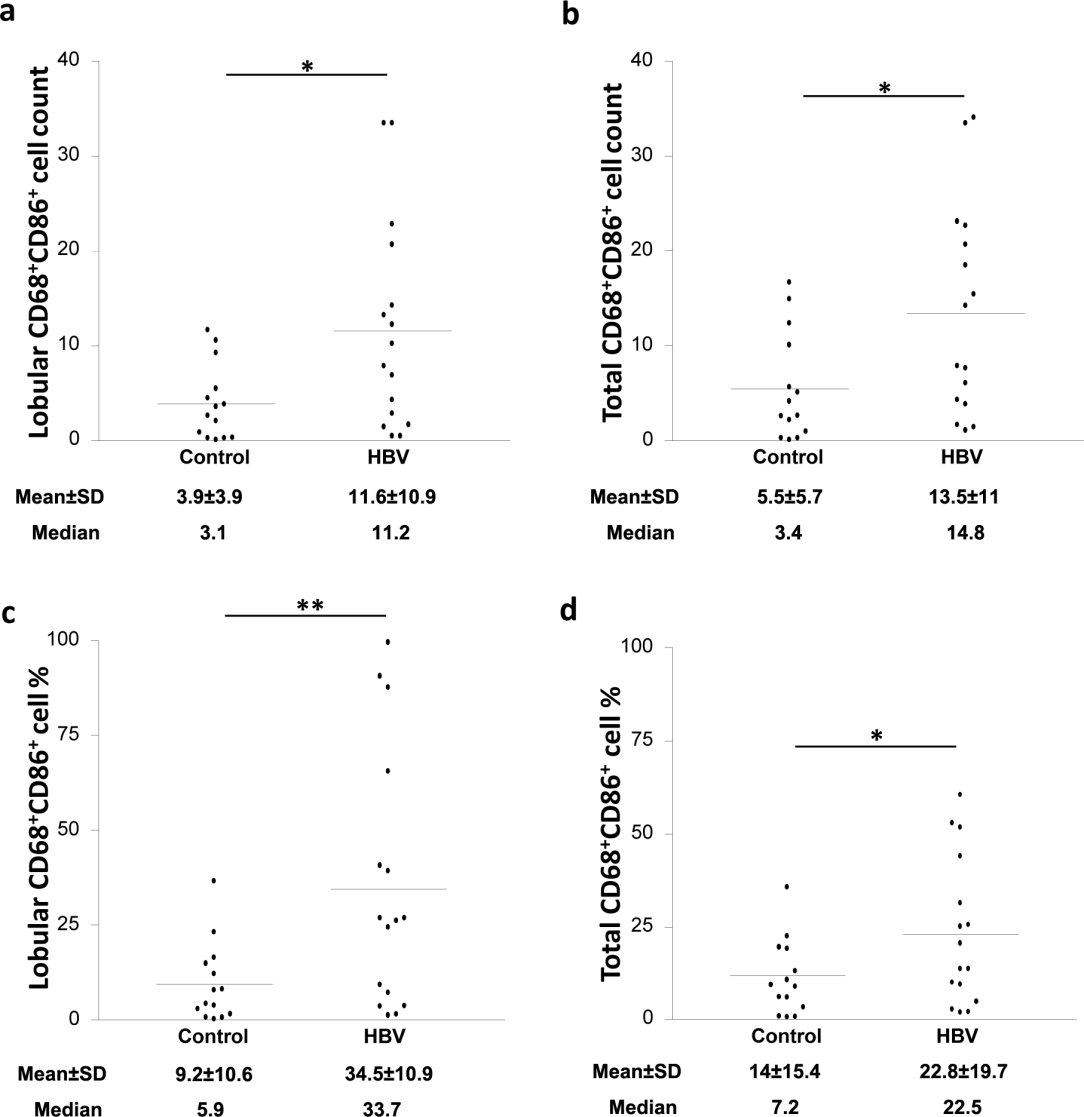
HBV infection is associated with elevated CD68^+^CD86^+^ cell count and percentage in the lobular areas of the liver. Biopsies from control and HBV-infected individuals were stained with mouse anti-human CD68 and rabbit anti-human CD86 Abs. CD68^+^CD86^+^ cell count in (**A**) the lobular area and (**B**) the total (lobular and portal) areas of the liver of control and HBV-infected individuals. CD68^+^CD86^+^ cell percentage in (**C**) the lobular areas and (**D**) the total (lobular and portal) areas of the liver of control and HBV-infected individuals. * *P* value<0.05. ** *P* value<0.01.

Macrophage ability to control the activation of T cells depends on their capacity to deliver costimulatory and inhibitory signals to T cells. The expression of the costimulatory molecules CD80 and CD86 on CD68^+^ cells was assessed using double staining based IHC (anti-CD68 Ab with anti-CD80 or anti-CD86 Abs). Interestingly, the count of CD68^+^CD86^+^ cells was significantly increased in the lobular areas of the liver of HBV-infected patients when compared to the control group (≈3-fold increase; *p* = 0.025; Fig 3A), whereas no significant difference was observed in the portal areas (Table 2). When the total CD68^+^CD86^+^ cell count (lobular and portal areas together) was calculated, HBV-infected patients showed a 2-fold increase when compared to the control group (*p* = 0.025; Fig 3B).

We further considered the percentage of CD68^+^CD86^+^ cells out of the total CD68^+^ cells. HBV-infected patients showed ≈4-fold increase in the percentage of CD68^+^CD86^+^ KCs in the lobular areas, but not the portal areas, when compared to controls *(p* = 0.0029; Fig 3C). The total percentage (lobular and portal areas together) were also calculated and HBV-infected patients showed ≈3-fold increase when compared to controls (*p* = 0.042; Fig 3D).

In contrast, no significant differences were found in the count and percentage of CD68^+^CD80^+^ cells in the lobular, portal and total areas when comparing HBV-infected patients to controls (Tables 2 and 3).

**Table 3:** Percentage of CD68^+^ cells, CD68^+^CD80^+^ cells, CD68^+^CD86^+^ cells and CD68^+^PD-L1^+^ cells. The average of the percentages ± S.D. for each areas of the liver is represented as indicated. The *p* value indicates the significance of differences between HBV-infected and control groups.

	Lobular	Portal	Total
	**CD68**^**+**^**CD80**^**+**^ **Percentage**		
**HBV-infected patients**	16.5 ± 18.5	20.8 ± 26.6	20 ± 47.9
**Controls**	13.7 ± 18.8	12.9 ± 15.8	12.1 ± 12.3
***P* value**	0.646	0.218	0.183
	**CD68**^**+**^**CD86**^**+**^ **Percentage**		
**HBV-infected patients**	34.5 ± 33.7	9.1 ± 13.2	22.8 ± 19.7
**Controls**	9.2 ± 10.6	10.5 ± 12.2	14 ± 15.4
***P* value**	0.0029	0.947	0.042
	**CD68**^**+**^**PD-L1**^**+**^ **Percentage**		
**HBV-infected patients**	1.5 ± 7.9	4.1 ± 3.2	1.2 ± 27.6
**Controls**	6.4 ± 5.6	3.9 ± 4.6	10.5 ± 8.8
***P* value**	0.827	0.964	0.561

Furthermore, the expression of the co-inhibitory molecule PD-L1 was assessed using a combination of anti-CD68 and anti-PD-L1 Abs. Our results showed no significant differences in the count and percentage of CD68^+^PD-L1^+^ cells in the lobular, portal and total areas when HBV-infected patients were compared to the control group (Tables 2 and 3).

We have observed that in the control group the count of CD68^+^CD80^+^ (*p* = 0.001; Table 2), CD68^+^CD86^+^ (*p* = 0.019; Table 2) and CD68^+^PD-L1^+^ cells (*p* = 0.001; Table 2) was higher in the lobular areas (cell density = 31.9±48.3 cells/mm^2^, 16.4±16.8 cells/mm^2^ and 10.4±8.4 cells/mm^2^ for cells expressing these molecules respectively) compared to the portal areas (cell density = 7.6±11.8 cells/mm^2^, 6.7±9.7 cells/mm^2^ and 2.1±3.4 cells/mm^2^ for cells expressing these molecules respectively). In HBV-infected patients, the count and percentage of CD68^+^CD86^+^ cells were higher (*p* = 0.003 and *p* = 0.01 respectively; Tables 2 and 3) in the lobular areas compared to the portal area (cell density = 48.7±45.8 cells/mm^2^ and 8±13.9 cells/mm^2^ for both areas respectively), and CD68^+^PD-L1^+^ cell count was higher (*p* = 0.001; Table 2) in lobular areas compared to portal areas (cell density = 9.2±8.4 cells/mm^2^ and 2.9±2.9 cells/mm^2^ for both areas respectively).

Of note, the count and percentage of CD68^+^ cells expressing CD80, CD86 or PD-L1 were neither correlated with HBV viral load (*p*>0.05), nor associated with the age and sex of the individuals, in the HBV-infected or control group (*p*>0.05).

### The correlation of CD80 expression with the fibrosis score and inflammation grade in HBV-infected liver

**Fig 4:**
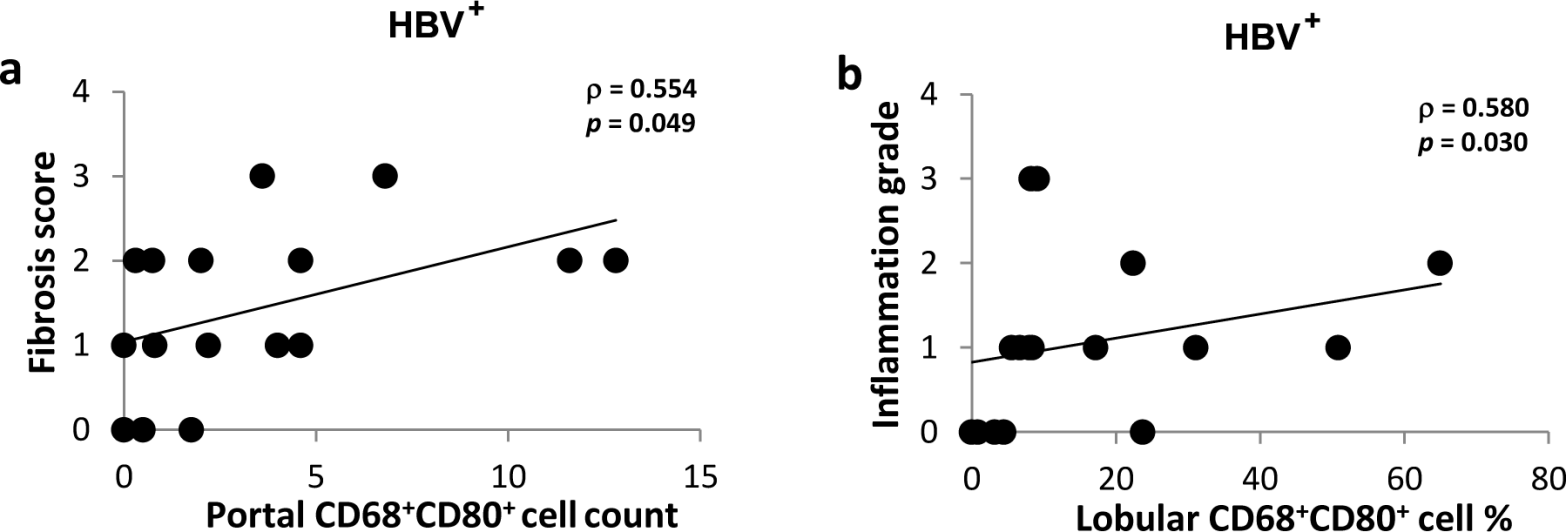
CD80 expression correlation with the fibrosis score and inflammation grade in HBV-infected liver. Biopsies from HBV-infected individuals were stained with mouse anti-human CD68 and rabbit anti-human CD80 Abs. Masson Trichrome stained slides were used to assess the fibrosis score and inflammation grade by Metavir scoring system. **A.** CD68^+^CD80^+^ cell count in the portal and the fibrosis score. **B.** The percentage of CD68^+^CD80^+^ cells in lobular areas and the lobular inflammation. * *P* value<0.05. ** *P* value<0.01.

We assessed the correlation of the costimulatory and inhibitory molecules expression with the degree of inflammation and fibrosis in the liver of HBV-infected patients. CD68^+^CD80^+^ cell count in the portal areas positively correlated with the fibrosis score (ρ = 0.554, *p* = 0.049; Fig 4A), but not with the inflammation grade (*p*>0.05). In addition, the percentage of CD68^+^CD80^+^ cells in lobular areas positively correlated with lobular inflammation (ρ = 0.580, *p* = 0.030; Fig 4B). No other correlations were found between the fibrosis score or inflammation grade and the count or percentage of CD68^+^CD80^+^, CD68^+^CD86^+^ and CD68^+^PD-L1^+^ cells (*p*>0.05).

## Discussion

Our results showed that chronic HBV infection did not alter the number of CD68^+^ cells in the liver. To our knowledge no other study reported on the count of CD68^+^ cells and Kupffer cells in the liver during HBV infection. The absence of increase in the count of these cells despite the presence of an active viral infection might be due to the interference of HBV with the pathways that lead to the attraction and activation of CD68^+^ cells and to the anti-inflammatory molecules expressed in these cells during HBV infection [7,29]. In this study CD68^+^ cells were mainly present within the lobular areas of the liver, precisely within sinusoids. This may be due to transendothelial migration, and is in line with earlier reports showing that the majority of KCs are present within the sinusoids in the lobule of the liver [30]. Nevertheless, another study that used CD163 as a marker observed a higher number of macrophages in the portal areas as compared to the lobules [31]. However, in contrast to CD68, CD163 expression is modulated by activation [32], which might impact the identification of macrophages in the liver based on CD163 expression. Interestingly, CD68^+^ cells distribution between the lobular and portal areas was not affected by HBV infection as it was similar to that in the control group.

This study is the first to assess the expression of CD80 and CD86 together with PD-L1 in liver of patients with chronic HBV infection using IHC-Double staining method. Interestingly, the count and percentage of CD68^+^ cells that expressed CD86 was higher in HBV-infected livers when compared to non-infected liver. Furthermore, in chronic HBV-infected patients, the percentage of cells expressing CD86 was higher in the lobular areas when compared to the portal areas. Although the count of CD68^+^CD86^+^ cells was higher in the lobular areas in comparison to the portal areas in both HBV-infected and control donors, this does not indicate an activation of CD68^+^ cells in the lobular areas in the control group as the total CD68^+^ cell count is also higher in the lobular areas in these individuals compared to the portal areas, and the percentage of CD68^+^CD86^+^ cells was similar between the two areas in the control group. These results indicate a higher activation of CD68^+^ cells in the lobular areas compared to the portal areas of the liver during chronic HBV infection, which corroborates with the results showing that detectable serum HBV DNA is associated with lobular inflammation [33].

On the other hand, the expression of CD80 and PD-L1 was not higher in HBV-infected patients compared to healthy individuals. A previous study found that only few KCs expressed CD80 and CD86 on KCs in HBV infection [22]. However, in that study KCs were identified base on their morphology only. While some studies showed that PD-L1 expression is increased on KCs during HBV infection [24,26], our results support studies showing no changes in the levels of PD-L1 expressed on KCs during HBV infection [23,25]. Of note, the study of Zhang et al. showed that PD-L1 upregulation on KCs was only when HBV viral load was <5x10^3^ copies/ml [26].

The presence of CD68^+^ cells that did not express any of CD80, CD86 or PD-L1 during HBV infection might be due to different reasons. This includes the lack of activation of some CD68^+^ cells because they were not exposed to HBV particles or because of their impaired activation upon uptaking HBV particles. This impairment can be due to the fact that some HBV proteins such as HBsAg and HBeAg, are able to interfere with toll-like receptors (TLRs) signaling that lead to APCs activation [34,35], or to the presence of molecules that can inhibit the expression of B7 molecules such as IL-10 and TGF-β, which are upregulated during HBV infection [7,36,37,38,39]. The exact reasons of the lack of CD80, CD86 and PD-L1 expression on these cells and whether they express other activation markers such as CD40 or PD-L2, for instance, remain to be investigated.

Different results were found in a related study about chronic hepatitis C virus (HCV) infection, as we observed an increase in the expression of CD80 and PD-L1 but not CD86 in CD68^+^ cells in the liver of the HCV-infected patients compared with the control group [27]. The different patterns of CD68^+^ cell activation might be due to the differences in the replication cycles and the structures of HCV and HBV, although both viruses replicate in hepatocytes.

There are differences in the way CD80 and CD86 expression is regulated, for example upon stimulation of DCs and macrophages, CD86 is upregulated within 6 hours and reach maximum levels at 18 to 24 hours; however CD80 is upregulated after 24 hours and reach maximum levels at 48 to 72 hours [40,41]. CD80 and CD86 expression is also differently regulated by several molecules such as cytokines and prostaglandins. Prostaglandin E2 (PGE2) inhibits the upregulation of CD80, but not CD86, upon phagocytes activation [42]. Interestingly, PGE2 expression is upregulated during HBV infection and this upregulation was linked to the presence of the HBV protein HBx [43]. Therefore, PGE2 might inhibit the upregulation of CD80 on CD68^+^ cells during HBV infection. The upregulation of CD86 but not CD80 on APCs is observed in other diseases as well such as systemic lupus erythematosus [44]. In contrast to CD80, CD86 expression on APCs drives the differentiation of T cell towards a Th2 profile [12,13,14,15,16]. Previous studies showed that in chronic HBV-infected livers, most T cells are Th0-like cells and contribute to the production of Th2-type cytokines by producing IL-4 and IL-5 in addition to a low level of IFN-γ production [2,17]. Therefore, the higher count and percentage of CD68^+^CD86^+^cells with the absence of an increase in CD80 expression might contribute to a shift of the T cell response towards a Th2 profile. An impaired production of Th1 cytokines and the counteraction of their effects by IL-4 produced by the majority of intrahepatic T cells in HBV infection may decrease the efficiency of the antiviral response, which necessitates a Th1 profile to be competent. In a transgenic mouse model of HBV replication, IFN-γ and TNF-α secreted by virus-specific cytotoxic T lymphocytes could clear HBV from the liver [17]. Moreover, the strength of Th1 responses was associated with the clearance of HBV infection, as these responses were stronger in patients who resolved their infection when compared to chronically infected patients [18]. However, the Th1 response was also associated with increased liver damage as the levels of HBV-specific TNF-α^+^ CD4^+^ T cells in the liver correlated with the degree of liver inflammation and fibrosis [18] and the levels of circulating TNF-α correlated with the degree of fibrosis [45]. Conversely, our results showed for the first time that the count of CD68^+^CD80^+^ cells in the portal areas correlated with the fibrosis score and the percentage of CD68^+^CD80^+^ cells in the lobular areas correlated with grade of inflammation in HBV-infected patients. This suggests that the expression of CD80 might contribute to the progression of fibrosis. This might be related to the role of CD80 in inducing TNF-α-producing Th1 cells. The number of CD68^+^ cells expressing CD80 is similar in HBV-infected patients and controls; however within the same group some individuals have more cells expressing CD80 than others, although all values are low. Therefore, in the presence of HBV antigens a higher number of Th1 cells will be potentially stimulated in patients who have more CD68^+^CD80^+^ cells [12,13,14,15,16]. The presence of a higher number of Th1 cells may lead to an increase in liver damage and the degree of liver inflammation and fibrosis [45]. Another hypothesis could be that PGE2, which is upregulated during HBV infection, is inhibiting CD80 upregulation [42,43], and supressing liver fibrosis and inflammation [46]. Therefore the levels of liver fibrosis and inflammation might be correlated with CD80 expression on CD68^+^ cells because both phenomena are influenced by PGE2. Moreover, CD80 has been found to be highly expressed on the M1 subset of macrophages as compared to other subsets [47]. CD181 (CXCR1), which is a receptor of IL-8, is also highly expressed on M1 macrophages [47]. IL-8 contributes to liver inflammation and fibrosis by increasing the accumulation of CD181^+^ macrophages in the liver [48]. Interestingly, the levels of IL-8 are increased during HBV infection [49], which suggests that IL-8 might be causing an accumulation of CD181^+^ macrophages in the HBV-infected liver. Of note, polymorphism in the *CXCR1* gene was found to be associated with the disease activity during chronic HBV infection [50]. Therefore, it is possible to hypothesise that CD80 expression on CD68^+^ cells might be associated with a higher expression of CD181 on these cells. This suggests that CD181 expression might differentiate the pattern of CD68^+^ cells in patients and controls and CD181 expression on these cells might correlate with the inflammation and fibrosis scores in HBV-infected patients. The inflammation and cellular damage associated with CD80 expression may affect the viral replication, as cellular viability is necessary for this replication, therefore the absence of CD80 upregulation on KCs is in the advantage of HBV replication.

In conclusion, the upregulation of CD86 but not CD80 and PD-L1 on CD68^+^ cells in the liver of HBV-infected patients, observed in our study, suggest that the profile of CD68^+^ cells does not support the induction of proper Th1 responses that are needed to clear HBV infection. This might provide an explanation for the absence of potent HBV-specific T cells during chronic HBV infection. These findings suggest that anti-HBV vaccinal and therapeutic strategies should consider the induction of an adequate CD80 expression on CD68^+^ cells in the liver of the patients.
